# A multimodal antimicrobial stewardship intervention to improve antibiotic prescribing in patients with COVID-19

**DOI:** 10.1017/ash.2023.462

**Published:** 2023-10-19

**Authors:** Shaylee Anderson, Nicholas Bennett, Laura Aragon, Kevin Kennedy, Sarah Boyd

**Affiliations:** 1 University of Utah, Salt Lake City, UT, USA; 2 Saint Luke’s Health System, Kansas City, MO, USA; 3 Saint Luke’s Hospital of Kansas City, Kansas City, MO, USA

## Abstract

This study assessed outcomes prior to and after electronic medical record-based clinical decision support implementation combined with prospective audit in patients with COVID-19. This multimodal stewardship intervention was associated with a decrease in antibiotic exposure for patients with COVID-19 (44.4% vs 61.8%, *p* = 0.002) within the first 7 days of hospitalization.

## Introduction

Respiratory tract infections (RTI) are a leading cause of hospitalization and death in the United States.^1^ Understanding that viral RTIs are more common than bacterial, empirically treating all patients with antibiotics leads to unnecessary antibiotic exposure, increasing risk of antibiotic resistance, cost, and adverse events.^1^ This was common practice with the emergence of COVID-19, however, studies conducted early in the pandemic found antibiotics were prescribed in 56%–72% of patients despite low bacterial co-infections (3%–8%).^2–4^ With this information, judicious antibiotic prescribing became a stewardship imperative.

Previous internal research demonstrated that implementation of an electronic medical record (EMR)-based provider best practice alert (BPA) significantly decreased antibiotic days of therapy.^5^ Modeled after this success, we replicated the process for patients with COVID-19. We concurrently implemented targeted prospective audit for patients with COVID-19 to identify opportunities to de-escalate antibiotics, representing a combined active and passive intervention.

## Methods

This was a pre-post, multi-site study at 7 hospitals within Saint Luke’s Health System (SLHS) and received investigational review board waiver approval. All data points were collected via chart review from the EMR, EPIC (Epic Systems, Verona, WI).

**Figure 1.**
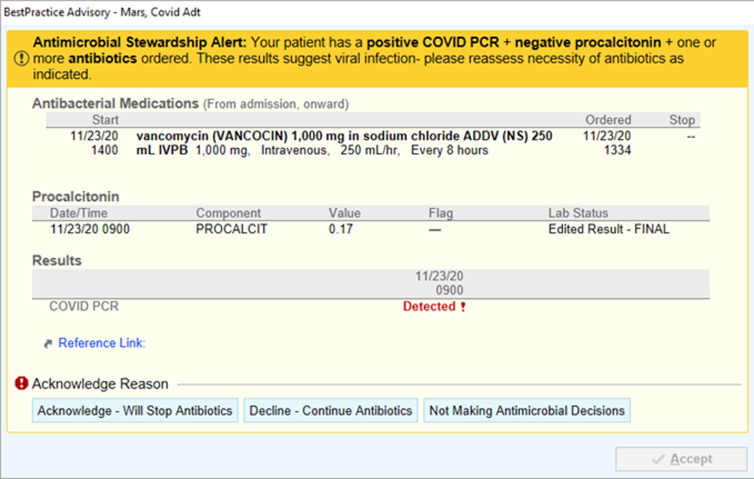
Best practice alert.

In November 2020, an automated BPA was implemented for all system hospitals. This alert fired upon any provider opening a patient chart when alert criteria were met encouraging antibiotic de-escalation with documented viral illness (Figure 1). The alert was triggered by a confluence of negative PCT (<0.25 ng/mL), positive COVID-19 PCR, and active antibiotic use. Concurrently, an antimicrobial stewardship program (ASP) pharmacist began actively identifying patients during prospective audit who had COVID-19 and were on antibiotics to identify opportunities for de-escalation. Antimicrobial stewardship program pharmacist efforts could have been in addition to or in the absence of a BPA firing on a patient (BPA criteria not met).

Patients were included in the study if they were 18 years or older and had a PCR-confirmed COVID-19 infection on admission. Patients were excluded if they transferred from outside of SLHS, the primary admission diagnosis was not COVID-19, or required antibiotics for a non-respiratory indication. The preintervention group included patients admitted in October 2020 and the postimplementation group included those admitted in January 2021.

The primary endpoint was percent of patients prescribed antibiotics within the first 7 days of hospitalization. Secondary endpoints include duration of antibiotic therapy (days), antibiotic days of therapy, length of hospital stay, bacterial co-infections on admission, *Clostridioides difficile* infection, re-initiation of antibiotics after discontinuation for any cause, percent of discharge antibiotic prescriptions, and inpatient mortality. Bacterial co-infections were confirmed based on positive blood or respiratory culture results. Blood and respiratory cultures were considered relevant if they were a possible respiratory pathogen and treatment was pursued based on provider documentation. A preplanned subgroup analysis assessed patients who had a BPA fire in the postimplementation group compared to patients in the preimplementation group who would have also met criteria.

## Statistical analysis

Data are shown as mean +/− SD or median (IQR) for continuous data and compared between pre- and postintervention cohorts using either the Student’s T-test or Wilcoxon Test. Data are shown as *n* (%) for categorical data and evaluated using chi-square tests. Data for the primary outcome was non-normally distributed data so we used the non-parametric alternative tests. All analysis was done with SAS 9.4 (Cary, NC).

## Results

This study included 298 patients (preintervention 136, postintervention 162). Baseline characteristics were similar between groups except for a higher postintervention Charlson Comorbidity Index score (1.9 vs 2.6, *p* = 0.039). There was no difference in confirmed bacterial co-infections (preintervention 8.8% vs postintervention 9.7%; *p* = 0.528). For the primary outcome, significantly fewer antibiotics were prescribed in the postintervention group in the first 7 days (44.4 vs 61.8%, *p* = 0.002). For secondary endpoints, the duration of antibiotic therapy within the first 7 days of hospital admission (2 vs 4 d, *p* = 0.034) and total antibiotic days of therapy (4 vs 6 d, *p* = 0.022) were significantly lower in the postintervention group. There was no difference between groups among other secondary endpoints (Table 1). A preplanned subgroup analysis of patients in the postintervention group that had a BPA triggered (*n* = 33) vs those in the preintervention group that would have met criteria for the BPA (*n* = 64) had a lower duration of antibiotic therapy (2 vs 3 d, *p* = 0.014) and antibiotic days of therapy (4 vs 6 d, *p* = 0.011).

**Table 1.** Primary and secondary results

	Total (*n* = 298)	Preintervention (*n* = 136)	Postintervention (*n* = 162)	*P*-value
**Primary Outcome**				
Antibiotic orders during first 7 days of admission (*n*[%])	156 (52.3%)	84 (61.8%)	72 (44.4%)	**0.002**
Secondary outcomes				
Duration of antibiotics (days) Median (IQR)	3.0 (2.0, 5.0)	4.0 (2.0, 6.0)	2.0 (2.0, 5.0)	**0.034**
Antibiotic days of therapy Median (IQR)	6.0 (4.0, 8.0)	6.0 (4.0, 8.0)	4.0 (3.0, 7.0)	**0.022**
Length of hospital stay (days) – Mean ± SD	7.2 ± 7.9	7.3 ± 7.2	7.1 ± 8.5	0.832
Inpatient mortality (*n*[%])	24 (8.0%)	10 (7.4%)	14 (8.6%)	0.683
*Clostridioides difficile* infections (*n*[%])	1 (0.3%)	0 (0%)	1 (0.3%)	0.362
Re-initiation of antibiotics after initial discontinuation for any reason (*n*[%])	23 (9.6%)	7 (8.4%)	16 (10.2%)	0.296
Antibiotics prescribed at discharge (*n*[%])	12 (4.1%)	6 (4.4%)	6 (3.9%)	0.806

Note. IQR, interquartile range; SD, standard deviation.

## Discussion

A multimodal intervention was implemented to address antibiotic over-prescribing in patients with COVID-19 by leveraging active stewardship through prospective audit coupled with EMR-based clinical decision support. Cumulative efforts resulted in improved antibiotic prescribing, highlighting the importance of intra-pandemic stewardship efforts. Our intervention was associated with decreased antibiotic exposure, including a decreased overall percentage (17.4%) of patients receiving antibiotics within 7 days of admission, duration of antibiotic therapy, and antibiotic days of therapy. Similar to previous studies, we found low rates of bacterial co-infections.^2–4^ A subgroup analysis for only patients that met BPA criteria found a 2-day decrease in antibiotic days of therapy and 1-day decrease in duration of antibiotics.

Limitations of our study included 2 comparator groups during 2 separate waves of the COVID-19 pandemic, which may have led to unforeseen confounding factors, including clinical management standards, which continuously evolved throughout the entirety of the pandemic. Given the rapidity of practice change, there was a concern that even greater bias may have been introduced if we had not compared populations close in time. Second, it is possible that antibiotic prescribing may have been impacted by factors outside of formal stewardship recommendations and/or BPAs leading to decreased prescribing. Third, the BPA was fired based on initial PCT results on admission, possibly at a time when PCT may not have peaked. However, we did assess antibiotic re-initiation and there was no difference between groups. Additionally, the study was limited to the first 7 days of inpatient admission and does not characterize subsequent effects on antibiotic use. Finally, we did not differentiate, nor stratify outcomes based on severity of illness.

Our study also had strengths. First, we allowed for an adaptation period between initiation of the BPA and more active ASP efforts in November 2020 to post-implementation data collection. This may have improved the clinical discernment related to BPA agreement when it triggered and/or ASP recommendations over time. Second, we limited the study population to only those with potential respiratory co-infections to provide a more accurate representation of attributable antibiotic use in the COVID-19 population, without skewing our results with other unrelated infections. Third, we replicated this practice after previous research demonstrating success using similar BPA strategies.

This study suggests that a multimodal antimicrobial stewardship intervention effectively decreased antibiotic use in patients with COVID-19. We found success leveraging a passive approach using BPAs and active ASP pharmacist efforts to further impact prescribing. However, there are multiple mechanisms by which decreasing antibiotic prescribing can be targeted. One study found success by creating an ASP-developed institutional guideline. Compared to our study, they found similar success in reducing antibiotic prescribing (32.5%) and antibiotic duration (1.3 d), highlighting the importance of institution-specific process design.^6^ These strategies could be replicated by other health systems to improve antimicrobial stewardship.

## Conclusion

For newly admitted patients with COVID-19, our study found leveraging ASP pharmacists and EMR alerts is associated with decreased antibiotic exposure leading to enhanced patient care.
